# Classification of Surface Deformation Using DTW-Transformer Model in Lower Jinsha River Basin

**DOI:** 10.3390/s26175515

**Published:** 2026-08-31

**Authors:** Mingbo Liu, Wei Wu, Wei Chen, Peng Han, Meiyu Liu, Shaoqiang Meng, Thomas Glade

**Affiliations:** 1 National Disaster Reduction Center of China, Ministry of Emergency Management of the People’s Republic of China, Beijing 100124, China; wuwei@ndrcc.org.cn (W.W.); hanpeng@ndrcc.org.cn (P.H.); liumeiyu@ndrcc.org.cn (M.L.); 2 School of Earth System Science, Tianjin University, Tianjin 300072, China; chenwei19@tju.edu.cn; 3 Department of Geotechnical Engineering, Tongji University, Shanghai 200092, China; mengs36@univie.ac.at; 4 Department of Geography and Regional Research, University of Vienna, 1010 Vienna, Austria; thomas.glade@univie.ac.at

**Keywords:** InSAR time-series analysis, Dynamic Time Warping, surface deformation in reservoir areas, Lower Jinsha River Basin

## Abstract

InSAR-based monitoring and classification of surface deformation are essential for the timely detection and mitigation of geological hazards in reservoir areas. However, the intricate effects of environmental factors, the nonlinear nature of deformation delays, and the overwhelming data volumes continue to impede the establishment of universal approaches for the rapid classification of InSAR-derived time-series surface deformation in reservoir areas. In this study, we developed a method combining Dynamic Time Warping (DTW) and a Transformer to process deformation data extracted from SBAS-InSAR. The proposed method uses the similarity derived from the DTW distance matrix as the training objective for the Transformer model. During inference, the trained model generates embeddings for the entire study area through a single forward pass, without requiring further pairwise DTW computation. We also combined prototype assignment with unsupervised clustering to identify different deformation patterns. The experiment was conducted in the Lower Jinsha River Basin, where newly constructed reservoirs have triggered extensive surface deformation responses. With DTW’s time-elastic alignment and Transformer’s self-attention mechanisms, our model better distinguished deformation time series than static similarity methods, while maintaining linear computational complexity with respect to sample size during inference. The proposed method identified different deformation patterns in the study area and found that their spatiotemporal characteristics exhibit complex relationships with water level fluctuations, precipitation, and lithology.

## 1. Introduction

The Jinsha River Basin in southwestern China has dramatic topographic relief and hosts several large hydropower stations that play a crucial role in clean energy production, regional economic development, and flood control. However, water level fluctuations caused by reservoir impoundment and regulation can affect slope stability in reservoir areas, posing risks to the safety of dams and hydropower facilities and increasing geological hazard threats for nearby communities. To mitigate these risks, large-scale and continuous deformation monitoring is required, along with systematic classification and evaluation of deformation characteristics [1,2,3].

Ground-based sensors provide continuous, high temporal resolution monitoring of deformation in critical areas [4,5]. Data acquired from drones and high-resolution optical satellites are widely used for landslide identification and establishing landslide inventories [6,7,8], forming the basis for landslide susceptibility studies [9,10,11,12,13]. However, these methods are limited in large-scale, long-term continuous monitoring due to issues such as data accessibility and coverage, and the ability to detect slow deformations. InSAR technology has advantages in this field due to its millimeter-level precision and ability to provide continuous observations under various weather conditions [14]. Several InSAR techniques have been developed, including conventional Differential InSAR [15,16], Small Baseline Subset (SBAS) InSAR [17,18], Permanent Scatterer (PS) InSAR [19,20], and SqueeSAR [21], all of which have been successfully applied to geological hazard monitoring. For example, SAR data from ALOS-1 and Sentinel-1 have been used to identify reservoir bank landslides in the Jinsha River Basin, and the spatial distribution of landslides, as well as their correlations with reservoir water levels and rainfall, have been analyzed [1,22]. Other studies have combined time-series InSAR data with geological structural features to examine the main triggering factors, deformation mechanisms and failure modes of individual landslides in the Wudongde Reservoir area [18,23].

Classification methods for InSAR time-series surface deformation have been widely explored. Early studies utilized simple linear regression models and deviation indexes to characterize InSAR time series [24,25]. Conditional statistical tests were employed to classify deformation, revealing that different trend patterns reflect distinct physical mechanisms driving slope motion [26]. This approach was later refined through the incorporation of trend classification and periodicity assessment [27]. Although these statistical approaches are effective for describing general deformation trends, they mainly rely on predefined statistical features and classification criteria, which may limit their ability to characterize complex and nonlinear deformation patterns. With the rise in machine learning and deep learning techniques, a broader range of methods has been applied. Principal Component Analysis (PCA) combined with K-Means was utilized to reduce dimensionality and identify clusters [28], while Density-Based Spatial Clustering of Applications with Noise (DBSCAN) and its hierarchical extension (HDBSCAN) were adopted to capture cluster structures [29,30]. However, these clustering methods do not explicitly account for temporal misalignment between deformation time series, which may reduce the measured similarity between sequences associated with the same deformation process but exhibiting different response delays. Consequently, using static similarity measures without considering elastic alignment can produce biased clustering results. The Long Short-Term Memory (LSTM) model was used to detect sudden velocity changes [31], and a hybrid framework combining Stacked AutoEncoders (SAE) with Convolutional Neural Networks (CNN) was proposed to classify ground subsidence [32]. More recently, a one-dimensional CNN integrated with self-supervised contrastive learning and rotation-consistency augmentation has been applied to categorize InSAR deformation time series into five classes: Phase Unwrapping Error (PUE), deceleration, linear, acceleration, and stable [33]. These methods provide stronger data-driven feature learning capabilities, but their ability to characterize long-range temporal dependencies may be limited by the underlying recurrent or convolutional architectures.

Dynamic Time Warping (DTW) finds the optimal alignment path between two time series by allowing nonlinear stretching and compression along the time axis, thereby accommodating the nonlinear characteristics of time-series surface deformation [34]. Transformers leverage self-attention to capture temporal dependencies in time series, enabling effective long-range modeling and representation of complex dynamic patterns beyond the capabilities of traditional Recurrent Neural Networks (RNNs) or CNNs [35]. By combining DTW and Transformer architectures for time-series deformation data, the proposed method takes advantage of both temporal alignment and self-attention, a synergy that has seldom been examined in prior studies. Furthermore, prototype assignment [36] and unsupervised clustering are incorporated to classify deformation and discover new patterns. Experiments were conducted in the Wudongde Reservoir area of the Lower Jinsha River Basin.

## 2. Study Area and Data

### 2.1. Study Area

**Figure 1:**
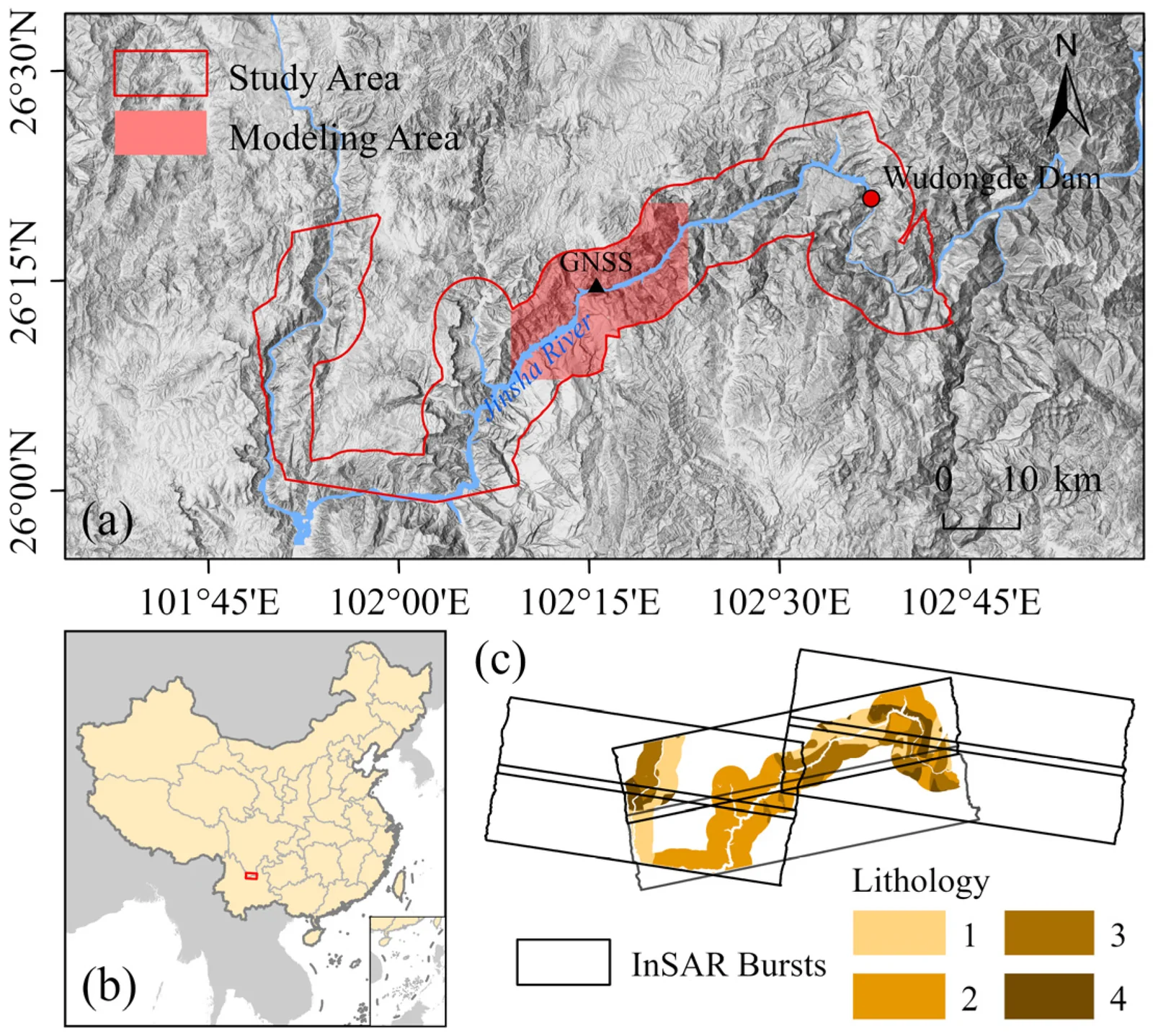
Study area, lithological groups, and InSAR data coverage. (**a**) Extent and topography of the study area and modeling area. (**b**) Location of the study area. (**c**) Lithological groups and coverage of the InSAR data. Explanations of the lithological groups are provided in Section 2.2.2.

The study area is located in the Wudongde Reservoir region of the Lower Jinsha River Basin (101.81–102.73° E, 25.96–26.45° N) (Figure 1a), with elevations ranging from 690 to 3250 m and a total area of 1625.32 km^2^. The northern part belongs to Huidong County in Sichuan Province, while the southern part lies in Luquan County in Yunnan Province (Figure 1b). Situated on the southeastern margin of the Tibetan Plateau, the area has a complex geological and geomorphological landscape, shaped by intense tectonic activity and river incision. The dominant lithologies include Quaternary conglomerates, sandstones, siltstones and clays, as well as Permian limestones, dolomites and basalts (Figure 1c). The vegetation is primarily pine and broadleaf forests, while below an elevation of 2000 m, extensive barren land is exposed, favoring landslide and debris flow development. The region has a subtropical monsoon climate with frequent rainfall from May to October, and dry conditions with sparse precipitation from November to April. This region is densely populated and affected by intensive engineering activities, making it highly susceptible to geological hazards. A 5 km buffer zone around the reservoir was selected as the study area, with a representative section in the central part chosen for model development.

The reservoir began impoundment in 2020, and its water level changes can be broadly divided into three stages. The first stage, from April to June 2020, raised the water level from 928 m to 950 m. The second stage, from July to September 2020, further increased it to 971 m. The third stage began in October 2020, during which the water level fluctuated above 950 m with multiple rises and falls. The most significant declines occurred from April to September 2021 and from June to October 2022.

### 2.2. Data

#### 2.2.1. SAR Dataset

Sentinel-1A is a C-band SAR satellite launched in April 2014. It acquires SAR images over the study area every 6 or 12 days from ascending and descending directions. The Sentinel-1 Single Look Complex (SLC) product is composed of three sub-swaths, each containing several bursts. Bursts have fixed footprints, and the smaller area of each individual burst significantly reduces computational and storage costs (Figure 1c). To select burst interferometric pairs while maintaining a sufficiently dense and connected interferometric network, the perpendicular baseline was constrained to less than 300 m, with a temporal baseline ranging from 12 to 36 days, an Interferometric Wide (IW) beam mode, VV polarization, and Ground Sample Distance (GSD) of 20 m [37]. A total of 328 ascending and 656 descending SLC bursts, with a temporal coverage from January 2018 to May 2023, were downloaded, respectively, from the Alaska Satellite Facility (ASF, https://search.asf.alaska.edu) for interferometric processing (Table 1).

#### 2.2.2. Lithology Dataset

Based on the 1:1,000,000 public geological map of the study area and China’s national standard for engineering classification of rock mass [22], the lithology of the study area was divided into four groups in order of increasing hardness, with Group 1 being the least hard and Group 4 the hardest (Table 2).

## 3. Methods

**Figure 2:**
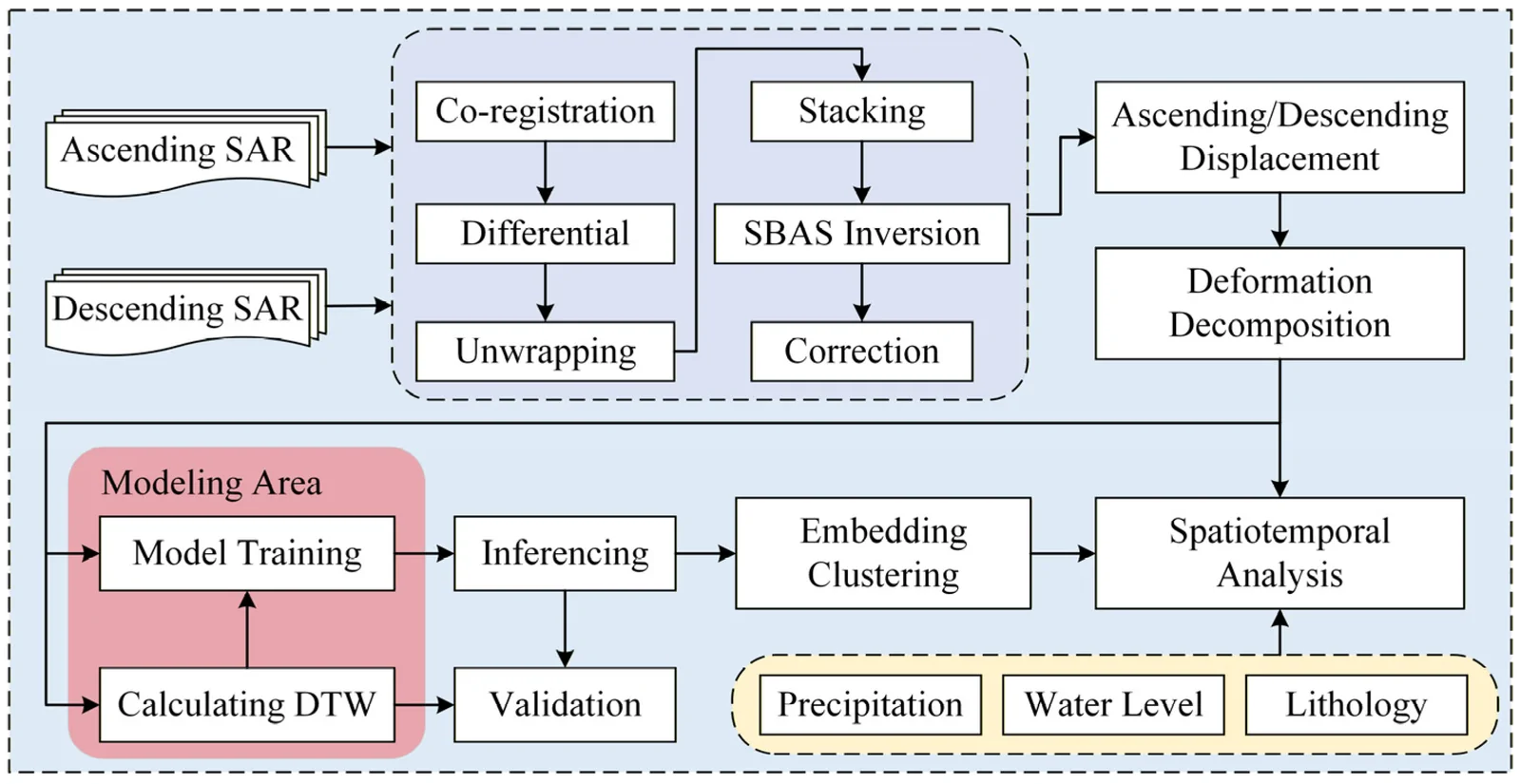
The flow chart of InSAR surface deformation classification and analysis. The model training was conducted only in the modeling area.

The workflow of this study is shown in Figure 2. First, the SBAS-InSAR method was applied to obtain Line of Sight (LOS) deformation sequences in both ascending and descending directions, followed by three-dimensional decomposition. Then, the vertical deformation time series within the modeling area were normalized, and the DTW distance matrix was calculated. After that, the deformation time series and DTW distance matrix were input into the Transformer model for multiple rounds of training and validation. The trained model was then used to process the deformation time series of the entire study area to obtain embeddings. Using prototype assignment and unsupervised clustering, all embeddings were grouped into clusters representing different deformation classes. Finally, the spatiotemporal distribution characteristics of different deformation classes were analyzed with environmental variables.

### 3.1. SBAS-InSAR and Time-Series Surface Deformation Extraction

SBAS-InSAR is a time-series InSAR inversion method based on multiple master images, which can effectively reduce spatiotemporal decorrelation and improve the accuracy of deformation monitoring. The main workflow consists of generating subsets based on temporal and spatial baselines, estimating the deformation phase of each subset with the least squares method, and obtaining deformation results through inversion. Open-source software ISCE2 v2.6.3 (https://github.com/isce-framework/isce2.git, accessed on 16 July 2026) was used for InSAR processing, with the GLO-30 Copernicus DEM as the topographic reference. MintPy (https://github.com/insarlab/MintPy, accessed on 16 July 2026) was employed for time-series processing, in which the interferogram stack of the original phase time series was inverted and deterministic phase components were corrected to obtain a noise-reduced deformation time series. The global atmospheric model ERA5 from the European Centre for Medium-Range Weather Forecasts (ECMWF) was applied to correct tropospheric delay, followed by topography and phase ramp corrections [38]. The resulting deformation time-series data have a spatial resolution of 20 m and a temporal interval of 12 days. Post-processing includes removing areas affected by layover and radar shadow using a layover-shadow mask generated from topography and imaging geometry. The ESA WorldCover land cover classification map was used to exclude forest and water pixels. Spatial high-pass filtering and temporal low-pass filtering were performed on the deformation time series to reduce residual atmospheric delays and decorrelation noise. In both Ascending and Descending datasets, pixels with a final cumulative deformation less than 3 cm were masked as noise, and connected regions containing no more than 25 remaining pixels were masked as insignificant deformation units. These two thresholds were empirically determined based on previous studies [18,22,37].

Since InSAR measures deformation along the LOS directions of ascending and descending tracks, studies often estimate three-dimensional surface deformation by either ignoring the north–south (N-S) displacement or using the surface-parallel flow assumption [39]. Following previous studies in the research area, we decomposed the LOS deformation from the ascending and descending tracks into east–west (E-W), N-S and vertical (U-D) components using the surface-parallel flow assumption. Specifically, the three-dimensional decomposition was calculated as follows:

(1) $$\left[\begin{array}{c} d_{as}\\ d_{des}\\ 0 \end{array}\right]=\left[\begin{array}{ccc} \cos\theta_{A} & -\sin\theta_{A}\cdot \sin\left(\alpha_{A}-\frac{3}{2}\pi \right) & -\sin\theta_{A}\cdot \cos\left(\alpha_{A}-\frac{3}{2}\pi \right)\\ \cos\theta_{D} & -\sin\theta_{D}\cdot \sin\left(\alpha_{D}-\frac{3}{2}\pi \right) & -\sin\theta_{D}\cdot \cos\left(\alpha_{D}-\frac{3}{2}\pi \right)\\ 1 & \tan\beta \cdot \cos\left(\frac{\pi }{2}-\lambda \right) & \tan\beta \cdot \sin\left(\frac{\pi }{2}-\lambda \right) \end{array}\right]\cdot \left[\begin{array}{c} d_{u}\\ d_{e}\\ d_{n} \end{array}\right]$$

where $d_{as}$ and $d_{des}$ are deformations of the LOS direction measured by ascending and descending data. $d_{u}$, $d_{e}$, and $d_{n}$ respectively represent the deformation in the U-D, E-W, and N-S directions. $\theta_{A}$ and $\theta_{D}$ are the incident angles of the sentinel-1 SAR data along ascending and descending tracks. $\alpha_{A}$ and $\alpha_{D}$ are azimuth angles of ascending and descending tracks. β and λ are the slope angle and aspect angle obtained according to the DEM, respectively. Subsequent modeling and classification were performed on the U-D component time series [23,40].

### 3.2. Dynamic Time Warping

In the DTW distance matrix, each DTW(x,y) represents the cumulative cost along the optimal alignment path between time series x and y, reflecting their similarity. The calculation is as follows [34]: First, the absolute difference between each pair of time steps (i,j) in x and y is calculated to obtain the cost matrix C. Then, following the principle of dynamic programming, the optimal path with the minimum cumulative cost is searched within the cost matrix. The optimal path to a given point is extended from the optimal path of its predecessor:

(2) $$A\left(i,j\right)=C\left(i,j\right)+min\left\{A\left(i-1,j\right),A\left(i,j-1\right),A(i-1,j-1)\right\}$$

where $A\left(i,j\right)$ represents the cumulative cost at position (i,j), and $C\left(i,j\right)$ represents the local cost $\left|x_{i}-y_{j}\right|$. The operator $min\left\{\cdot \right\}$ indicates that at each step the path with the minimum cumulative cost is chosen, and the indices cannot move backward but only progress forward. Finally, the cumulative cost at the endpoint of the optimal path is DTW(x,y).

### 3.3. Time Series Similarity Measures

In this study, the time series similarity measures involved are DTW similarity ${sim}_{DTW}$, embedding similarity ${sim}_{Emb}$, and Euclidean distance similarity ${sim}_{Euc}$. For a pair of time series x and y, the three similarities are calculated as follows:

(3) $${sim}_{DTW}\left(x,y\right)=exp\left(-\frac{DTW(x,y)}{\tau_{DTW}}\right)$$

(4) $${sim}_{Emb}\left(x,y\right)=exp\left(-\frac{\left\|E_{x}-E_{y}\right\|}{\tau_{Emb}}\right)$$

(5) $${sim}_{Euc}\left(x,y\right)=exp\left(-\frac{\left\|x-y\right\|}{\tau_{Euc}}\right)$$

where E represents the embedding vectors obtained from the model. The dynamic scaling parameter τ is defined as the median of the upper triangular part of each similarity matrix. Another commonly used similarity measure is cosine similarity, but for normalized time series, Euclidean distance and cosine distance exhibit a near-linear relationship and can be regarded as equivalent in clustering tasks.

### 3.4. Transformer and Multi-Head Self-Attention

**Figure 3:**
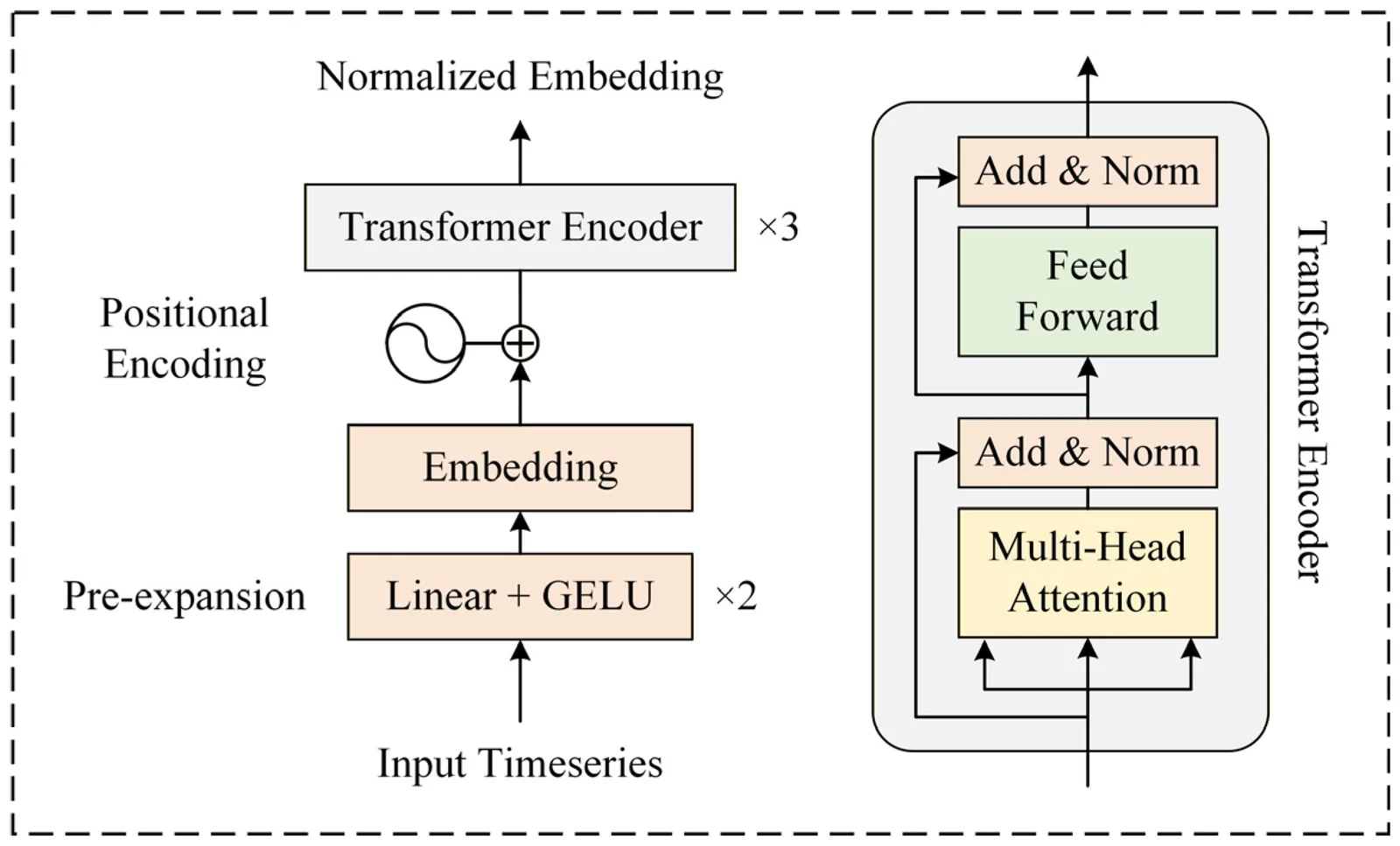
Transformer architecture used in this study. The Transformer Encoder architecture is illustrated on the right separately. Our model incorporates three Transformer Encoder blocks.

The Transformer architecture used in this study is shown in Figure 3 [35]. The input is the normalized deformation time series. A pre-expansion layer with two linear transformations, each followed by a GELU activation, increases the dimension to 16. The embedding layer then converts the features to 64 dimensions. Learnable positional encoding is added to include temporal information. Each encoder consists of 8 self-attention heads, a feed-forward network, residual connections, and layer normalization. The final output is the normalized embedding vector.

We designed a similarity loss function to guide the model toward DTW-based similarity. In the loss function, the Euclidean distance between embeddings is calculated and transformed into similarity ${sim}_{Emb}$. The true similarity ${sim}_{DTW}$ is obtained from the pre-computed DTW distance matrix. The difference between the predicted similarity and the true similarity is penalized by squaring, and a hard mining strategy is applied, where only the top 30% of sample pairs with the largest errors are selected for optimization. By focusing optimization on informative difficult samples, the hard mining strategy improves training efficiency and robustness, while the 30% selection ratio was chosen as a practically motivated choice rather than a theoretically optimal value. Model parameters are initialized with Xavier uniform distribution, the optimizer is Adam, and the learning rate is set to 0.003. Training is performed on a GPU. In each epoch, the samples are randomly shuffled, batches of size 256 are fed into the model, the loss is computed, and parameters are updated by backpropagation. The converged model weights are then saved. In addition, the attention weights are used to observe which time steps the model focuses on when processing different time series:

(6) $$Query\left(Q\right)=XW_{Q},\;Key\left(K\right)=XW_{K},\;Value\left(V\right)=XW_{V}$$

(7) $$Attention\left(Q,K,V\right)=softmax\left(\frac{QK^{T}}{\sqrt{d_{k}}}\right)V$$

For an embedding vector X, linear transformations are applied to obtain the three components Query, Key, and Value. *W* is a trainable weight matrix, which is updated during training through backpropagation and gradient descent and $d_{k}$ is the dimension of each attention head.

### 3.5. Prototype Assignment and Unsupervised Clustering

The advantage of using prototype assignment is that it incorporates prior knowledge to ensure physical meaning and improves robustness through the use of an assignment threshold [36]. The prototype assignment process consists of the following steps: A global threshold was first applied to detect noise and exclude unreasonable deformation time series, and the remaining data were then normalized. The model weights trained in the modeling area were loaded, and inference was performed to obtain the embedding vector of each time series. Five deformation classes were labeled [33]: accelerating subsidence (AS), continuous linear subsidence (CS), stable (ST), uplift (UP), and decelerating subsidence (DS), with 30 samples in each class. For every class, the mean of the embedding vectors was calculated to obtain the prototype embedding, which represents the centroid of that class in the embedding space. The mean ($\mu_{c}$) and standard deviation ($\sigma_{c}$) of the distances between the sample embeddings and the prototype embedding were calculated, from which the assignment threshold was derived:

(8) $${AT}_{c}=\mu_{c}+k_{\sigma }\cdot \sigma_{c}$$

where $k_{\sigma }$ is the tolerance parameter, which controls the strictness of the assignment. Finally, the Euclidean distance between each sample embedding in the study area and the prototype embedding of every class was computed. Each sample was assigned to the class of its nearest prototype. If the nearest distance exceeded the assignment threshold, the sample was classified into the “other” category.

For the “other” category, unsupervised clustering was further applied to subdivide the samples, enhancing the ability to explore and distinguish complex temporal patterns. To meet the clustering requirements of large-scale time series, the Mini-Batch K-Means method [41] was used. The silhouette score was adopted as the evaluation metric for clustering performance. The final number of unsupervised clusters was determined by jointly considering the silhouette score and expert knowledge.

## 4. Results

### 4.1. Validation of InSAR Time-Series Deformation Using GNSS

**Figure 4:**
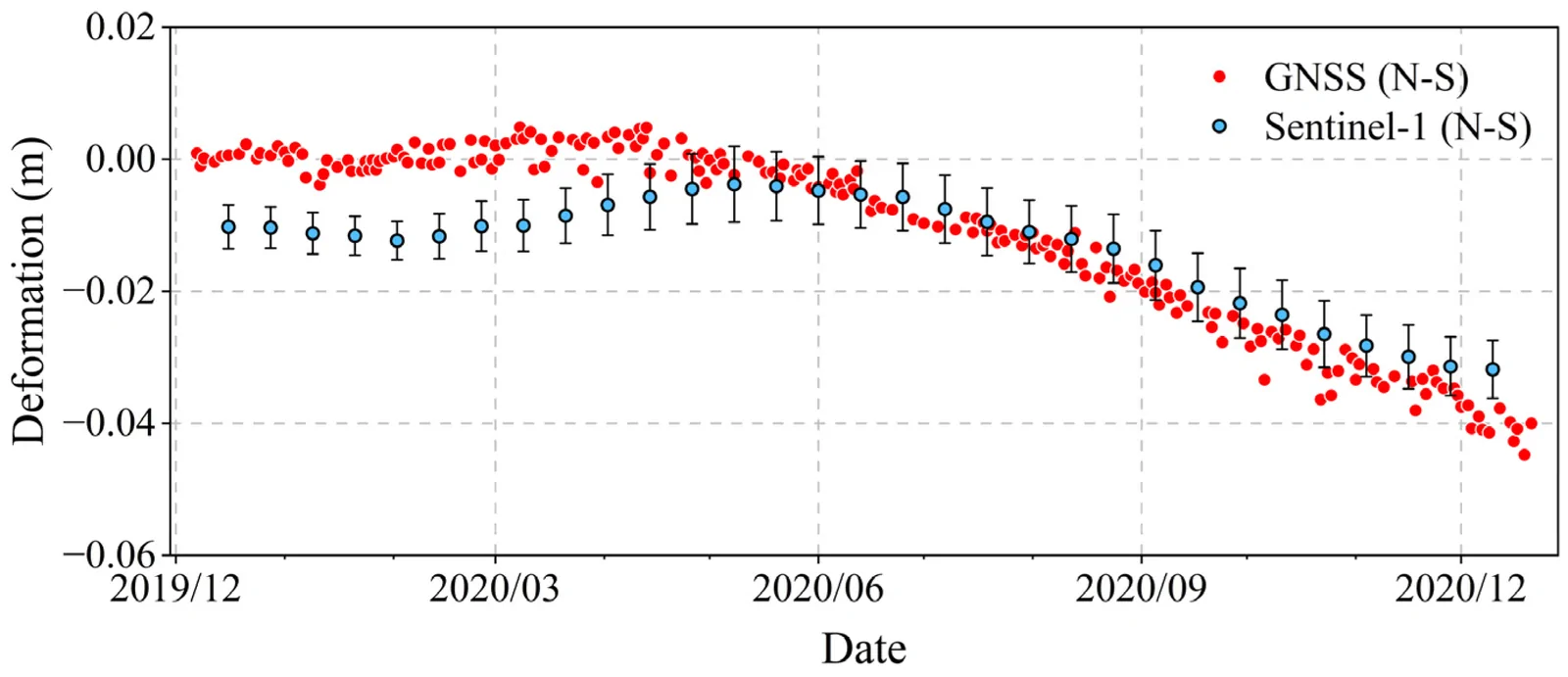
Validation of cumulative InSAR time-series deformation using GNSS data. The InSAR deformation values were represented by the median of the deformation within a radius of three pixels around the GNSS station site. The error bars correspond to the standard deviation within this range.

A GNSS station located in the central part of the study area provided continuous deformation monitoring data of the Zaogutian landslide during 2020 [23]. Since GNSS measurements are limited in detecting subtle vertical deformations and the landslide at the station site is oriented in the N-S direction, we compared the N-S deformation measured by the GNSS station with the N-S component of the InSAR deformation, as shown in Figure 4. To reduce the influence of geolocation bias and random errors, the InSAR deformation values were represented by the median of the deformation within a radius of three pixels around the station site. The error bars correspond to the standard deviation within this range [23]. As can be seen from the figure, although the InSAR time-series data before May 2020 were slightly affected by phase unwrapping errors and deviated from GNSS measurements, the two datasets remained strongly correlated, especially during the deformation stage.

The validation is limited by the availability of only one GNSS station and therefore provides a point-based assessment of temporal consistency rather than comprehensive spatial validation.

### 4.2. Performance of the Transformer Model

**Figure 5:**
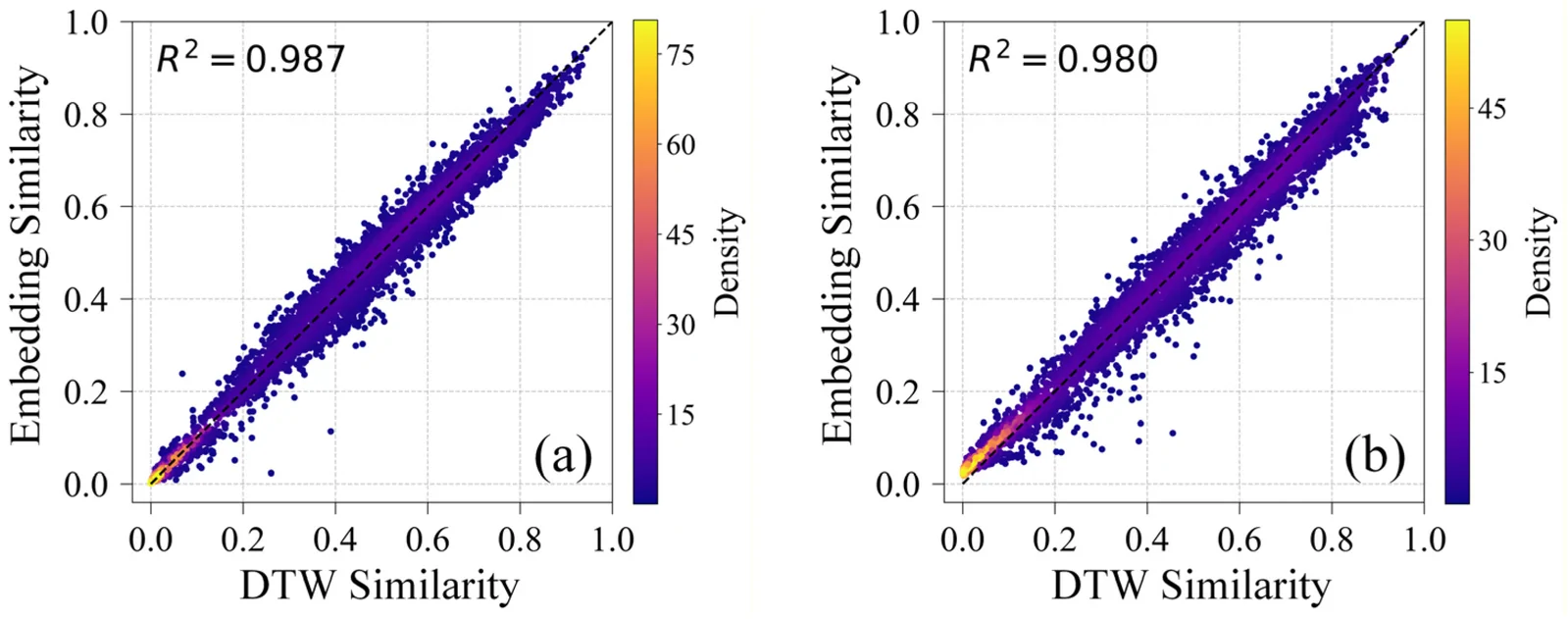
Comparison between embedding similarity and DTW similarity. (**a**) Within the modeling area. (**b**) Outside the modeling area.

**Figure A1:**
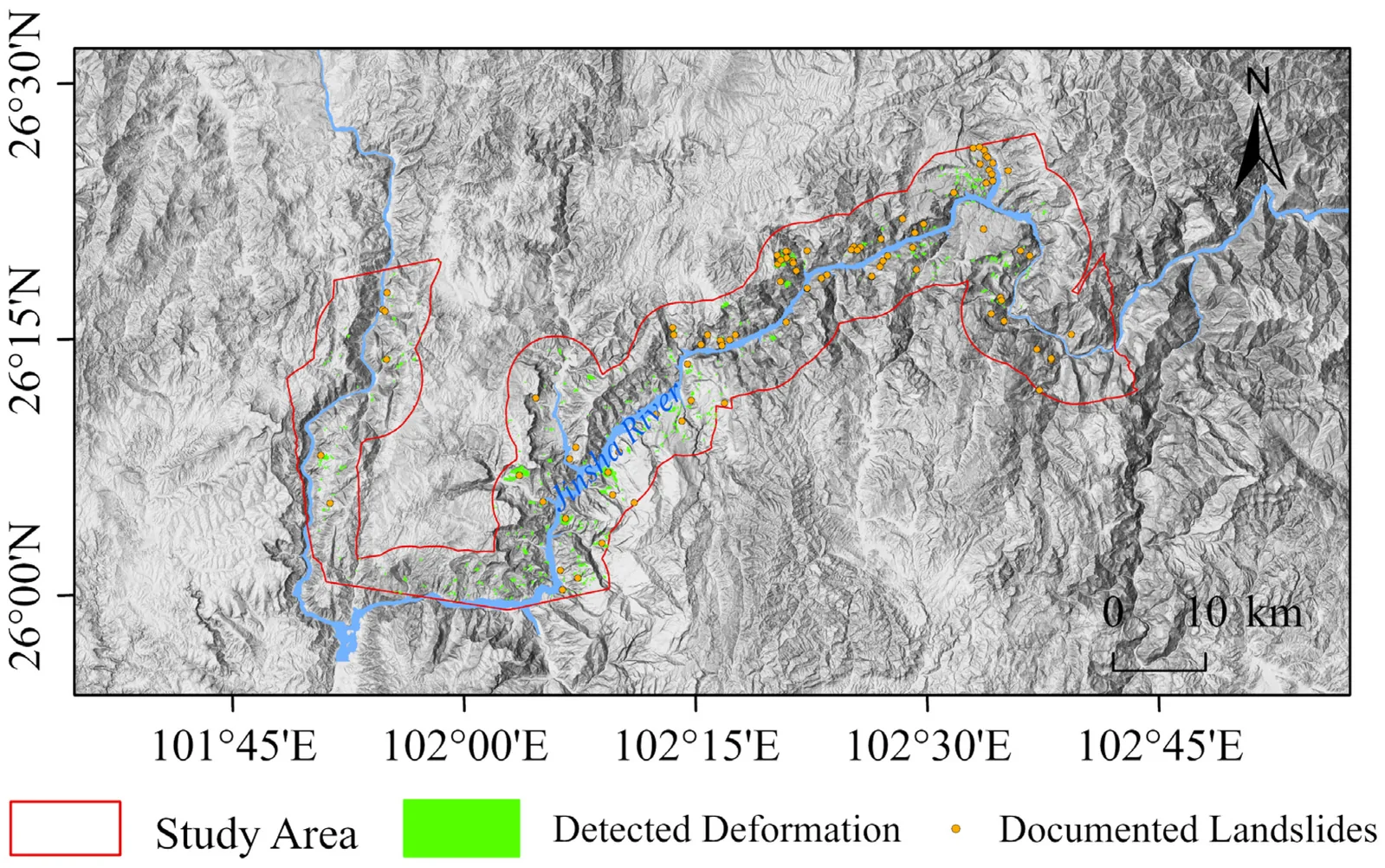
Detected significant deformation patches and documented landslides.

A total of 48,976 significant deformation pixels in 516 patches (Figure A1) were identified and classified in the study area, with each pixel representing a time-series of deformation. Of these, 13,196 pixels located within the modeling area were used for training. During the inference stage, the time-series data of all 48,976 pixels were processed. We randomly selected 100 samples within the modeling area to construct 4950 sample pairs, and compared their DTW similarity with embedding similarity, yielding an $R^{2}$ of 0.987 (Figure 5a). To further assess model performance beyond the modeling area, another 100 samples were randomly selected outside the modeling area for the same comparison, resulting in an $R^{2}$ of 0.980 (Figure 5b).

### 4.3. Temporal Characteristics of Different Deformation Patterns

**Figure 6:**
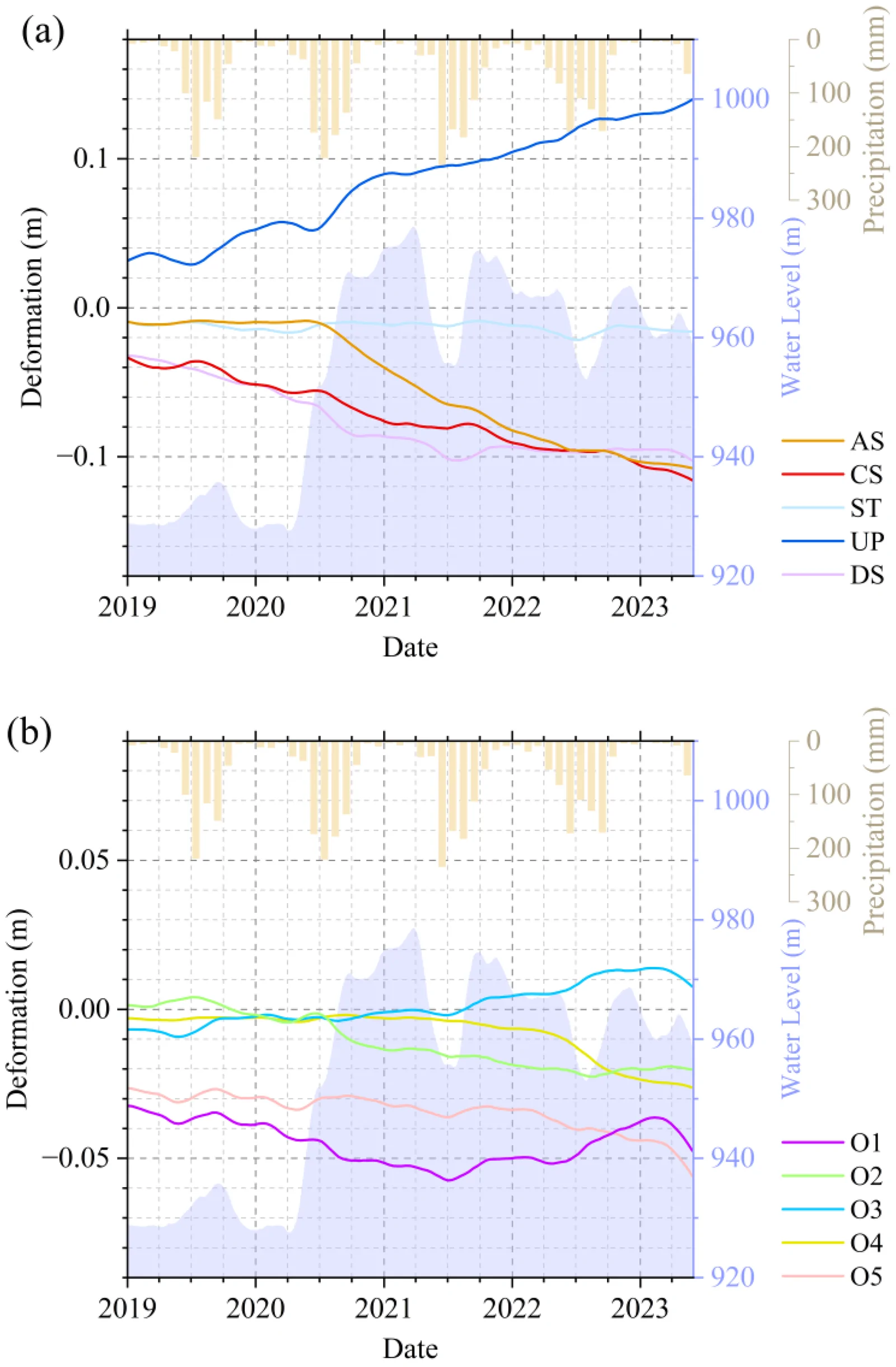
The deformation time series, reservoir water levels, and precipitation between January 2019 and May 2023. (**a**) Time series of five prototype classes. (**b**) Average time series of the five classes obtained by unsupervised clustering. Since the reference date of deformation is January 2018, not all curves in the figure start around zero.

Figure 6a,b present the time series of five prototype classes and the average time series of the five classes obtained through unsupervised clustering, where the number of clusters was determined by jointly considering clustering quality and the need for finer temporal characterization, resulting in a silhouette score of 0.342. Their temporal characteristics are summarized in Table 3. Different deformation classes exhibit distinct response patterns to triggering events, which likely result from internal stress redistribution or delayed pore water pressure dissipation, and are influenced by lithology and distance from the river.

### 4.4. Spatial Distribution of Different Deformation Patterns

**Figure 7:**
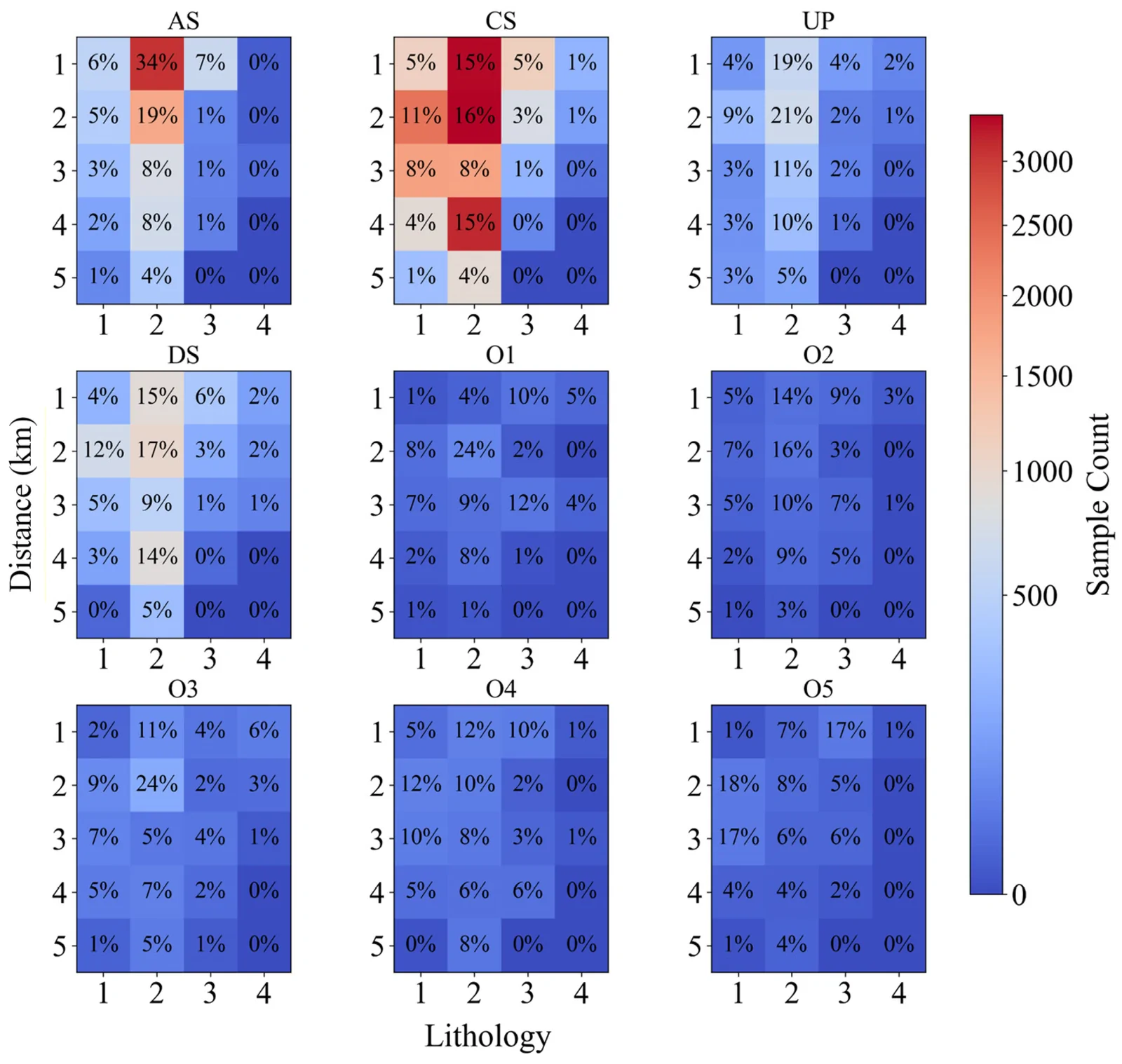
Distributions of different deformation classes across regions with varying distances from the river and lithologies. The percentage in each cell represents the proportion of samples under a given distance-lithology combination relative to the total number of samples of that deformation class. The color scale was nonlinearly stretched to highlight the distribution of classes with small sample sizes.

The distributions of different deformation classes across regions with varying distances from the river and different lithologies were compared (Figure 7). Even though lithology groups differ in area and position, certain distinct patterns can still be identified. It can be seen that most AS samples are distributed in a Moderately Weak lithology group (73.8%) and at a distance of less than 1 km from the river (47.1%), which partly explains why this class is most sensitive to reservoir water level changes. Compared with AS, the proportion of CS samples in the Weak lithology group is higher (29.9%), though the majority are still in the Moderately Weak lithology group (58.0%). Unlike AS, CS samples are most frequently found at a distance of 1–2 km from the river (30.6%). These lithology and distance features are clearly related to the typically larger deformation of this class and its weaker response to water level changes. UP samples usually correspond to loose deposits downslope of CS samples, and thus they show similar distributions. DS samples also show distribution patterns similar to CS and UP classes. O1 class has more samples distributed in the Moderately Hard lithology group and in areas farther from the river. The proportion of O2 samples in areas close to the river (31.7%) is second only to AS samples, which matched their temporal feature of short-term deformation mainly triggered by reservoir impoundment. O3 has a distribution similar to the UP class. O4 resembles AS with accelerating subsidence, but more samples (71.8%) are located at a distance of more than 1 km from the river, which may explain why their accelerating subsidence occurs two years later than AS samples. O5 is the only class mainly distributed in the Weak lithology group (40.8%), but being at a distance of more than 1 km from the river, they are barely affected by reservoir water levels.

### 4.5. Deformation Classification Map

**Figure 8:**
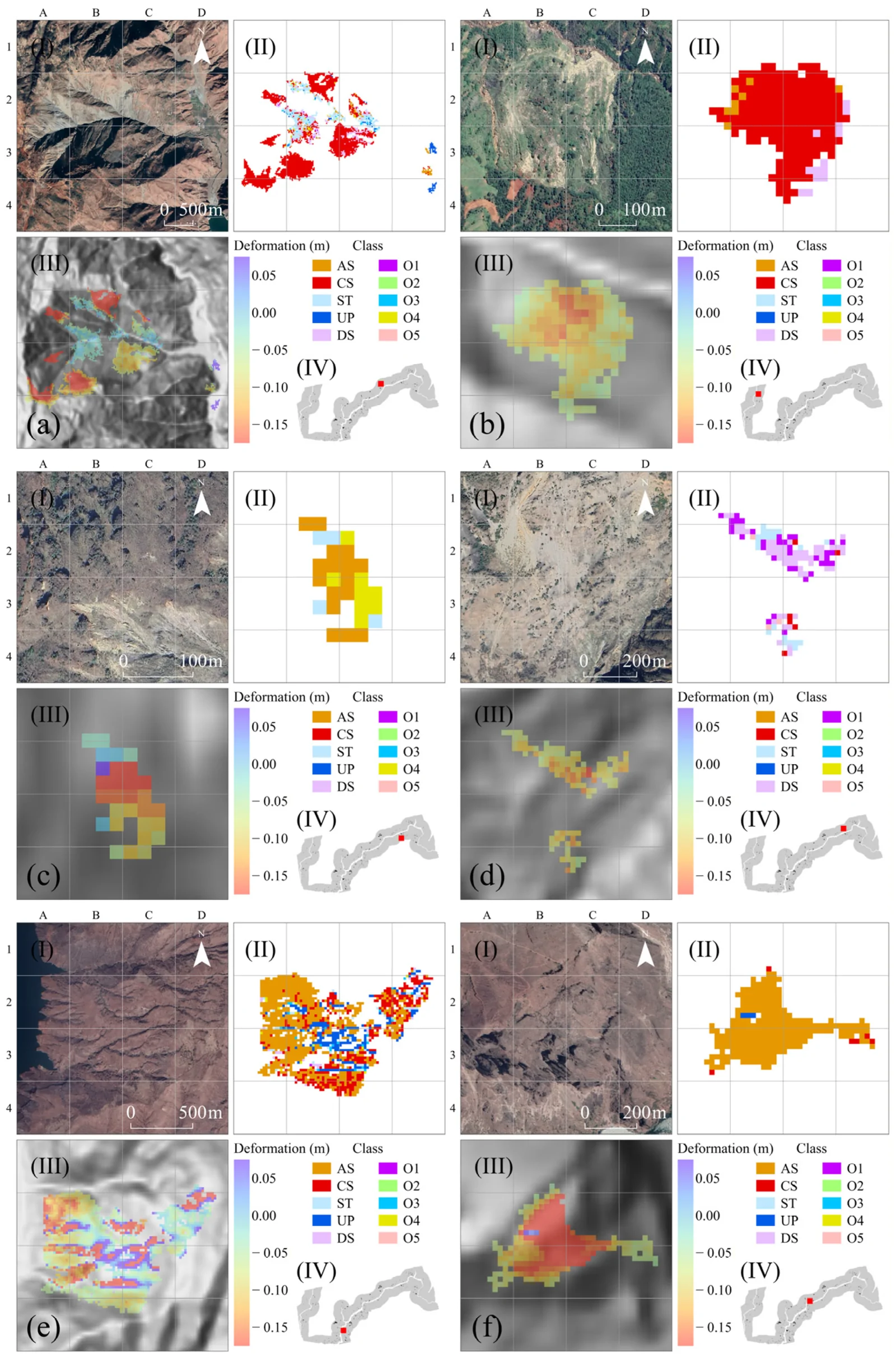
(**a**–**f**) Several representative regions of significant deformation. (**I**) Optical satellite image. (**II**) Classification map. (**III**) Cumulative deformation with hillshade. (**IV**) Location within the study area. All extracted significant deformation regions are also overlaid in (**IV**) and shown as black dots.

Figure 8a shows a landslide cluster. In the Google Earth image (I) obtained in December 2021, typical features such as debris-flow gullies and discontinuous boundaries can be seen. The deformation in this area is influenced not only by geological, hydrological, and topographic factors, but also by mining (e.g., C2) and road construction (e.g., A2). From the cumulative deformation map (II), it can be seen that the deformation is greatest on the surrounding slopes. The classification map (III) reveals more details. Large peripheral deformation zones (e.g., A3, C3), despite differences in magnitude, are mainly characterized by linear deformation. In contrast, the central areas (e.g., B2, C2), though showing smaller deformation, exhibit more complex patterns, including continuous linear subsidence (CS), decelerating subsidence (DS), stable (ST), and uplifts (UP). In some places, exposed bedrock prevents landslide movement. In region D3, we can find accelerating subsidence (AS) near the river, as well as uplift zones (UP) formed by material accumulation. Figure 8b shows deformation within a single landslide unit. The left edge displays an accelerating trend (AS), while the right edge shows a decelerating trend (DS), indicating the potential direction of future expansion. Figure 8c illustrates a landslide located away from the river, characterized by accelerating (AS) and delayed accelerating (O4) subsidence. Figure 8d shows a decelerating (DS) deformation zone away from the river. Figure 8e,f both depict landslides near the river dominated by accelerating (AS) deformation. Specifically, the Biyouzhao landslide in Figure 8e is an earthen slope with good permeability. During reservoir water level rise, seepage increases the slope’s weight, while the soil matric suction gradually decreases, leading to accelerated sliding at the front edge [22]. In Figure 8f, the Zaogutian landslide has well-developed fissures. The front edge is affected by scouring, soaking, and dynamic water pressure caused by reservoir water level fluctuations, which reduce the anti-sliding force and result in accelerated deformation [23].

## 5. Discussion

### 5.1. Comparison of Different Similarity Measures

**Figure 9:**
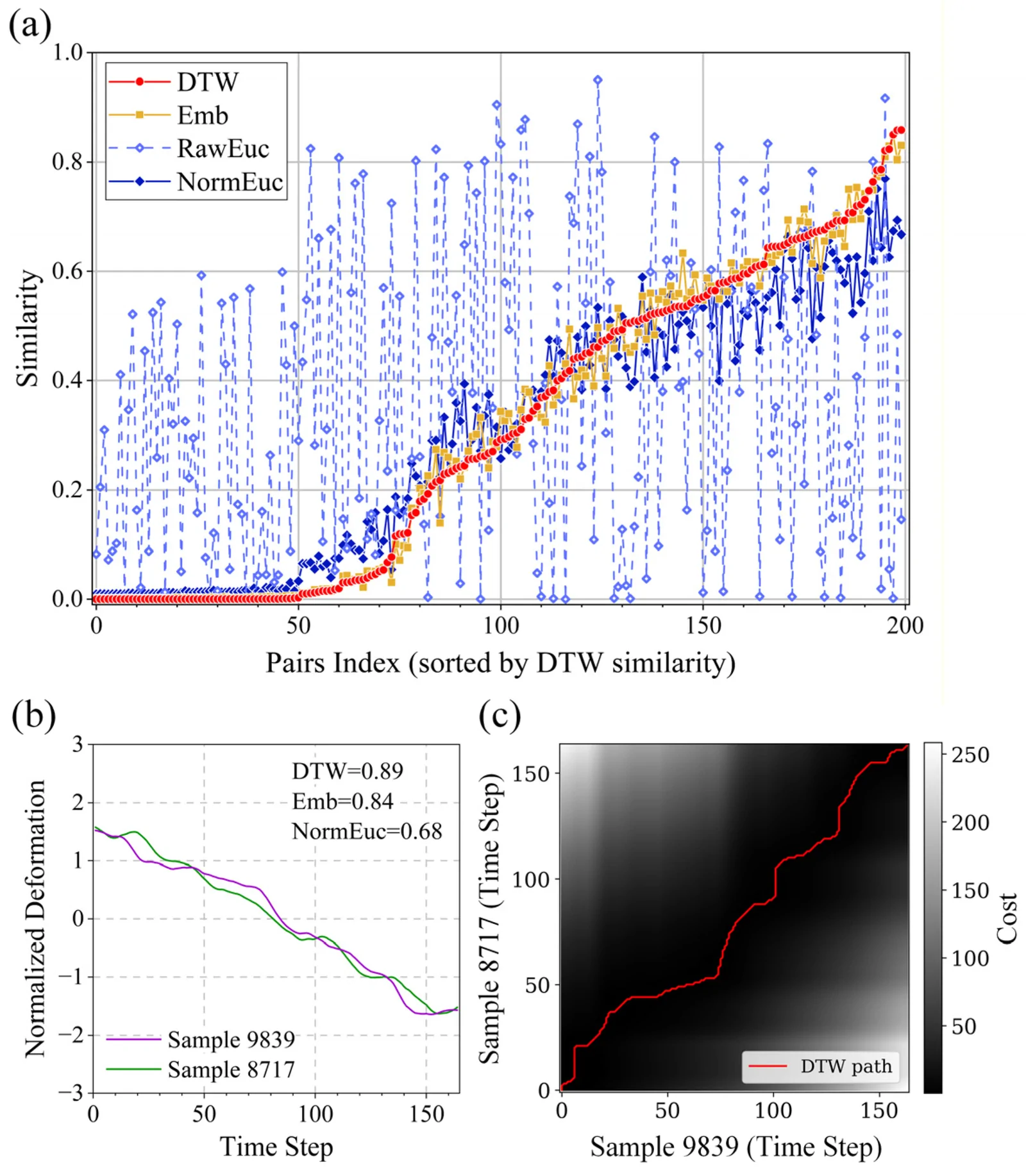
Different similarity measures for 200 randomly selected sample pairs, along with a demonstration of misalignment. (**a**) Comparison of different similarity measures. (**b**) Two time series with similar but temporally misaligned deformation patterns, annotated with their DTW, embedding (Emb), and normalized Euclidean distance (NormEuc) similarity. (**c**) The cost matrix and optimal DTW alignment path for the sample pair in (**b**).

We randomly selected 200 sample pairs and computed four similarity measures: ${sim}_{DTW}$ (DTW), ${sim}_{Emb}$ (Emb), and ${sim}_{Euc}$ for both raw (RawEuc) and normalized (NormEuc) time series (Figure 9a). The Euclidean similarity of raw time series is scattered due to amplitude variations, making it insufficient for fine-grained discrimination of most sample pairs. Embedding similarity is highly consistent with DTW, indicating effective model training. The Euclidean similarity of normalized time series is also close to DTW but shows greater variability. Notably, it underestimates some pairs with high DTW and embedding similarity.

Figure 9b illustrates the temporal misalignment using samples 9839 and 8717 as examples. Their normalized deformation time series show similar acceleration and deceleration patterns, but temporal misalignment of velocity changes produces point-by-point discrepancies, resulting in low Euclidean similarity. Applying DTW for elastic alignment along the time axis (Figure 9c) reveals higher similarity. Embedding similarity, obtained by feeding the two time series into the trained model, is consistent with DTW similarity (Figure 9b).

### 5.2. Attention Weight Visualization

**Figure 10:**
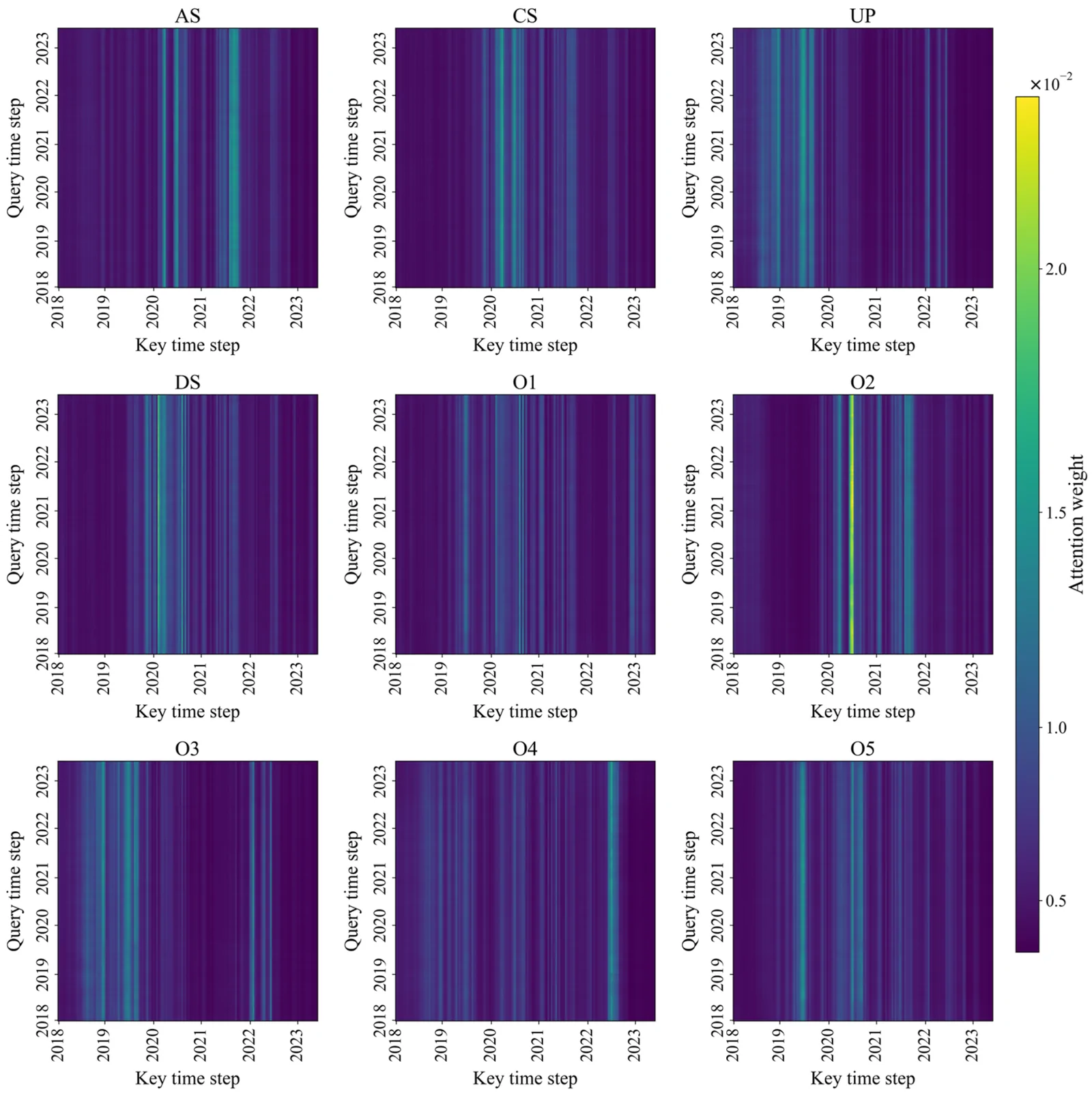
Average attention weights for different deformation classes. The bright regions highlight important temporal dependencies in the deformation time series.

The average attention weight matrix is obtained by averaging the weights across all attention heads, providing an overall attention pattern that reflects the global dependency structure at a given layer. We visualized the average attention weights in the final layer of the model during inference for different deformation classes (Figure 10).

For the AS class, high-weight periods occur on 2 April 2020, 7 July 2020, and between 7 August and 6 October 2021, corresponding, respectively, to the initial impoundment stage, the second impoundment stage, and the rising phase following the first drawdown from a high water level. The CS class exhibits high attention weights throughout the entire impoundment period, but with a more dispersed distribution, similar to the DS class. The UP class shows high-weight positions associated with precipitation peaks. The O2 and O4 represent two additional categories in which attention weights clearly correspond to water level changes. Specifically, the O2 class shows high weights from 13 June to 19 July 2020, corresponding to the transition from the end of the first impoundment stage to the beginning of the second stage, and from 7 August to 6 October 2021, corresponding to the rising phase after the first drawdown from a high water level. The O4 class shows high-weight positions corresponding to the second drawdown from a high water level. In contrast, the O1, O3, and O5 classes do not exhibit clear correspondence between attention weights and major stages of water level change, though they may be influenced by precipitation.

### 5.3. Ablation Study

**Figure 11:**
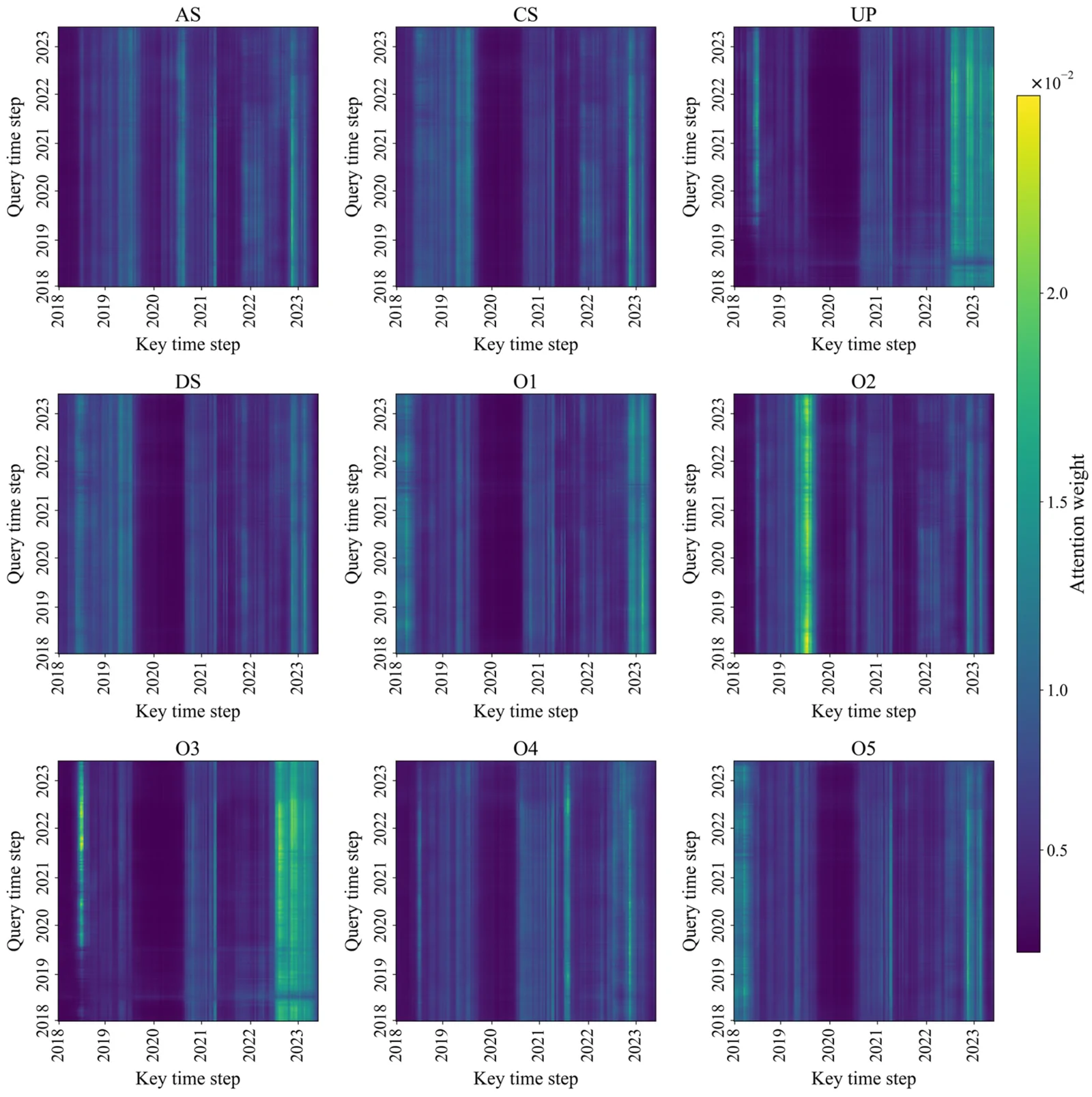
Attention weights after replacing the training objective with normalized Euclidean similarity. The bright regions highlight important temporal dependencies in the deformation time series.

To clarify the contribution of DTW, we replaced DTW similarity with normalized Euclidean similarity (i.e., Euclidean distance similarity for normalized time series) as the training objective and retrained the model, then visualized the attention weights (Figure 11). In the model trained with Euclidean similarity, the attention of all different classes became more dispersed, with significantly reduced focus on impoundment stages and greater emphasis on individual shape characteristics. This highlights the importance of DTW in model training, as it not only provides elastic alignment but also directs the model to focus on triggering events when distinguishing different deformation types, making the results more consistent with physical interpretation.

### 5.4. Computational Complexity

The computational cost of DTW increases steeply with data scale. Given a sample size of *N*, all pairwise distances between sequences must be computed, leading to a complexity proportional to $N^{2}$, i.e., $O(N^{2}\cdot L^{2})$, where L represents the sequence length. In contrast, inference with the trained Transformer model requires a single forward pass, and its complexity grows linearly with the sample size, i.e., $O(N\cdot \left(L^{2}\cdot d+L\cdot d^{2}\right))$, where d represents the embedding dimension. For large-scale InSAR deformation time series involving a large number of pixels, the Transformer model provides substantially faster computation than DTW, while maintaining high fitting accuracy, as confirmed by our experiments.

## 6. Conclusions

In this study, we developed a Transformer-based method using a similarity measure derived from DTW to classify and analyze deformation time series in large reservoir regions. Similarities derived from the learned embeddings show strong consistency with DTW-based similarities both within and beyond the modeling area. Experiments in the Wudongde reservoir area demonstrate that the proposed method performs better than static similarity measures such as cosine similarity and Euclidean distance when comparing deformation time series with temporal misalignment. This improvement is attributed to the temporal elastic alignment capability of the training objective and the model’s focused attention on triggering events. The inference speed of the Transformer model is also significantly faster than that of DTW. By integrating prototype assignment with unsupervised clustering, the method can identify known deformation types and reveal previously unknown deformation features. The deformation classification map, combined with deformation magnitude or rate maps, enables more detailed assessment of site-specific influencing factors and evolutionary trends.

Limitations include data gaps induced by decorrelation, a lack of GNSS coverage across diverse regions, and a requirement for field validation of deformation classes detected by unsupervised classification to exclude spurious signals from noise. For future research, we aim to mitigate these drawbacks and evaluate a range of deep learning architectures for comparative analysis.

## Figures and Tables

**Table 1:** SAR data used in this study.

Orbit Direction	Beam Mode/Polarization	GSD (m)	Number of Bursts	Temporal Coverage
Ascending	IW/VV	20	164 × 2	19 January 2018–29 May 2023
Descending	IW/VV	20	164 × 4	21 January 2018–31 May 2023

**Table 2:** Lithology groups classified by hardness.

Index	Lithology Group	Representative Rock Types
1	Weak	Phyllite, Slate, Schist
2	Moderately Weak	Mudstone, Shale, Clastic Rock
3	Moderately Hard	Sandstone, Carbonate Rock, Conglomerate
4	Hard	Amphibole–Plagioclase Granulite, Quartz Diorite, Granite

**Table 3:** Temporal characteristics of different deformation patterns.

Class	Characteristics
AS (Accelerating Subsidence)	Strongly influenced by reservoir water level changes. Accelerated two months after impoundment (April 2020), slowed with a 1–2 month delay when the water level decreased, and accelerated again as it rose.
CS (Continuous Linear Subsidence)	Overall linear downward trend, less affected by water level changes. Periodic minor deceleration and acceleration influenced by precipitation.
ST (Stable)	Negligible deformation throughout the study period.
UP (Uplift)	Overall linear upward trend. Strongly affected by precipitation before impoundment, and afterward evolved into steady uplift.
DS (Decelerating Subsidence)	Linear subsidence before impoundment, accelerated during impoundment, then decelerated. After second acceleration–deceleration cycle, slowed significantly, tending to stabilize.
O1 (Other 1)	Downward trend until June 2021. Subsidence stopped, and uplift occurred when water level decreased.
O2 (Other 2)	Relatively stable before impoundment, accelerated subsidence during the second impoundment stage, then decelerated, and tended toward stability after 2022.
O3 (Other 3)	Minor uplift, with acceleration in July of 2019, 2021, and 2022.
O4 (Other 4)	Stable until 2022, accelerated subsidence when the water level dropped from a high stage for the second time, and then stabilized again.
O5 (Other 5)	Slow fluctuating downward trend, minor influence of water level, and acceleration moments coincided with rainfall peaks.

## Data Availability

Data are available on request due to restrictions.

## Appendix A

A total of 516 significant deformation patches were detected in this study, of which 112 were associated with 91 documented landslides [18,22,37]. In addition, numerous previously undetected significant deformation patches were identified along both banks of the Jinsha River between Zhima and Jintaizi Village (Figure A1).
